# Coping strategies among adolescents with chronic headache and mental health problems: a cross-sectional population-based study

**DOI:** 10.1186/s40064-015-1599-x

**Published:** 2015-12-22

**Authors:** Silje Hartberg, Jocelyne Clench-Aas, Ruth Kjærsti Raanaas, Christofer Lundqvist

**Affiliations:** Department of Landscape Architecture and Spatial Planning, Norwegian University of Life Sciences, Aas, Norway; Health Services Research Centre, Akershus University Hospital, Lørenskog, Norway; Department of Neurology, Akershus University Hospital, Lørenskog, Norway; Institute of Clinical Medicine, Campus Akershus University Hospital, University of Oslo, Nordbyhagen, Norway; Division of Mental Health, Department of Health Surveillance and Prevention, Norwegian Institute of Public Health, Oslo, Norway

**Keywords:** Mastery, Tension type headache, Migraine, Young adults/students, Strengths and difficulties questionnaire

## Abstract

To examine prevalence of mental health problems among adolescents with chronic headache and compare internal and external coping strategies in young people with chronic headaches with and without mental health problems. This study is based on a cross-sectional survey undertaken in Akershus County in Norway. A total of 19,985 adolescents were included in the study, covering lower secondary and upper secondary students, aged 13–19 years. Chronic headache was measured with a single item question based on headache frequency. Mental health was assessed by using the strengths and difficulties questionnaire (SDQ). Internal and external coping strategies were assessed through seven options for answering the question: What do you do/what happens when you are burdened by painful thoughts and feelings? Adolescents with chronic headaches showed more frequent mental health problems overall (23 %) compared to those without chronic headache (6 %). Logistic regression analyses showed that those adolescents having both chronic headaches and comorbid mental health problems more frequently used internal coping strategies, such as keeping feelings inside (OR 2.05), using abusive substances (OR 1.79) and talking oneself out of problems (OR 1.55), compared to those without mental health problems. Groups with mental health problems, especially with chronic headache, less frequently used the external strategy of talking to others about their problem than controls (OR 0.7–0.8). Factor analyses revealed significant differences in profiles of coping strategies between groups. We suggest that attention should be paid towards the high risk group that has both chronic headaches and mental health problems and their tendency to use destructive internal coping strategies.

## Background

Headache disorders are among the top ten causes of disability in Europe (Steiner and Martelletti 2007; Steiner et al. 2015). Chronic headache is a major problem in children and adolescents (Gladstein 2004; Guidetti et al. 2000). It is characterized by a high degree of psychiatric comorbidity (Guidetti 2002). Stovner and colleagues (2007) found that the worldwide prevalence of chronic headache in the adult population was 3 % [2.4 % in Norwegian adults (Stovner et al. 2006)]. In children the prevalence ranges from 0.9 to 7.8 % worldwide (Seshia et al. 2010).

For children and adolescents who experience headache problems, psychological issues are well-recognized, but poorly understood clinical phenomena (Powers et al. 2006). The presence of psychiatric comorbidity is associated with poorer prognosis, decreased quality-of-life (Baskin et al. 2006) and increased psychosocial problems (Powers et al. 2006). Several studies suggest a bi-directional relationship between the comorbidity of headache and psychiatric disorders (Gentili et al. 2005; Wang and Juang 2002). Pompili and colleagues (2010) found that the relationship between migraine and psychopathology has not been systematically studied and suggested that future research should focus on the interplay of factors behind the relationship between migraine, suicide risk and mental illness. In the general population, on the other hand, because of relative low frequency of chronic migraine (Krogh et al. 2015), it is more interesting to study the relation between tension type headache and mental illness. However, studies suggest that psychopathology may be more linked to frequency of headache than to a specific headache diagnosis (Blaauw et al. 2015).

Coping has been defined as “constantly changing cognitive and behavioural efforts to manage specific external and/or internal demands that are appraised as taxing or exceeding the resources of the person” (Lazarus and Folkman 1984). It is described as “an ongoing dynamic process that changes in response to the changing demands of a stressful encounter or event” and as a “purposeful response […] directed towards resolving the stressful response between the self and the environment (problem focused coping) or toward […] negative emotions that arise as a result of stress (emotion-focused coping)” (Lazarus and Folkman 1984). In addition to this description, coping strategies have been described along axes such as “internal–external”, “voluntary–involuntary”, “engagement–disengagement” and “primary–secondary” (Lazarus and Folkman 1984; Compas et al. 2001). Though there is disagreement regarding the most constructive way to describe different ways of coping, some strategies have, nevertheless, been suggested to be more related to good mental health than others (Holen et al. 2012). This may be of importance regarding both diagnosis and treatment of disorders related to mental health and handling of stress and pain. The present study utilizes an internal vs. external coping strategy axis system (Lazarus and Folkman 1984; Pratt et al. 1985). This is based on the notion that some external strategies may be more strongly related to good mental health, as they are based on the individual seeking solutions outside him/herself e.g. through communication seeking external help in identifying and solving the problem. Internal strategies, on the other hand, involve efforts to regulate the emotional distress through internal efforts e.g. by venting negative emotions, using drugs/alcohol in attempts to attenuate the experienced distress and denying the association between the stressor and the experience (Jorgensen and Dusek 1990). Some internal strategies may be perceived as similar to avoidance strategies or withdrawal, which have been suggested to represent poor adaptation. This has been observed mainly in children and adolescents with depressive or anxiety symptoms (Holen et al. 2012; Chan 1995; Seiffge-Krenke 2000). An additional reason for choosing this categorization is that, in a treatment perspective the use of external strategies may be more accessible for therapy while internal coping may be more difficult to assess and influence.

In the field of headache, it is well established that chronic and frequent headache is associated with depressive symptoms and symptoms of anxiety as well as with more stressful life events (Wittrock and Myers 1998). In addition, headache patients have been suggested to use more maladaptive coping strategies (Wittrock and Myers 1998). Such coping patterns may especially involve the more internal coping strategies of avoidance and dissimulation (Wittrock and Myers 1998; Rollnik et al. 2001). Regarding coping patterns among youngsters with headache, studies have suggested such maladaptive strategies to be used more commonly among children and adolescents with headaches (Lanzi et al. 2001). Experimental studies have demonstrated that internalising strategies such as avoidance lead to increased distress while distraction, representing a more externalising coping behaviour decreases the level of distress, however, there are gaps in knowledge on coping and mental health problems in chronic headache among adolescents [(Compas et al. 2001) for review].

The present study aims to describe the prevalence and impact of chronic headache and mental health problems in adolescents using a well validated scale, the strength and difficulties questionnaire (SDQ) among a large representative (N = 19,985) sample of adolescents living in Norway. In addition, the aim was to compare the coping strategies favored by the different groups within an internal and external coping strategy framework.

## Methods

### Design and participants

This cross-sectional health survey was undertaken in Akershus County, Norway, an area including urban, suburban and rural areas, with clear differences in socio-economic status among the inhabitants.

Whole classes of pupils were selected to participate from randomly selected classes and schools in the county. The study included a total of 19,985 pupils from lower secondary school (n = 9414) and upper secondary school (n = 10,571), aged 13–19 years. The total response percentage was 82. Questionnaires were filled out at school, under the supervision of the teacher. A letter asking for parental consent with one reminder was sent to parents, prior to the study. The pupils that were invited to the study but did not participate, were primarily either home from school, on a school-trip or their teacher was off work.

### Measures

Four health groups were defined based on the two dependent variables chronic headaches and mental health problems. The groups were: “chronic headaches without mental health problems” (CH), “chronic headaches with simultaneous mental health problems” (CHMH), “mental health problems without chronic headaches” (MH) and a control group with neither chronic headache, nor mental health problems. The statistical analyses were done as a multinomial logistic analysis, with presence of each of the above defined health groups set as the dependent variable.

*Chronic headache* was assessed by the question “During the past 6 months, how often have you had the following complaints”, where headache is included as one of the complaints. The response possibilities were “almost every day”, “more than once a week”, “about every week”, “about every month”, “seldom or never”. “Almost every day” was defined as chronic headache in close accordance with the definition of chronic headaches according to the International Classification of Headache disorders, version 2 with chronic headache defined as more than half of the days with headache (Olesen and Steiner 2004).

*Mental health problems* were assessed using The strengths and difficulties questionnaires (SDQ) (Goodman 2011). We used four of the five original SDQ symptom scales, each with five items: emotional, conduct, hyperactivity and peer problems. The question about headache symptoms in the emotional subscale was excluded to avoid confounding the exposure (headache) and the outcome (SDQ). Each item has a three-point response scale (0 = not true, 1 = somewhat true, 2 = certainly true). Responses were rated 2 to 0 for positively worded items, and inversely coded for negatively worded items. The three subscales with five items each were summed to get a maximum total score of 10, whereas the emotion subscale with the headache question removed, summed to a maximum of 8. A total difficulties score was thus calculated based on adding the first four subscales scores, giving a total ranging from 0 to 38. It has previously been recommended to define three population groups (Goodman 2011); normal (lowest 80 % of population), borderline (10 %) and abnormal/caseness (highest 10 %). Further, Van Roy (2008) redefined the cut-offs to correspond to Norwegian symptom reporting, keeping the suggested 80-10-10 distribution. Since we removed one question from the SDQ, we redefined cut-off points for the normal group as 0–15, borderline scores from 16 to 19 and the abnormal group with scores from 20 to 38, corresponding as close to the Norwegian 80-10-10 cut-offs as possible (Van Roy et al. 2008). These values were for logistic regression further dichotomised into normal versus borderline/abnormal, which is a standard method of analysis (Goodman 2011).

To assess the impact of the mental health problem in everyday life, the extended version of the SDQ was used including five questions concerning overall distress and social impairment. Responses were coded into 0 = no/little, 1 = quite a lot, 2 = a great deal. The five items generate an impact score, ranging from 0 to 10. A total impact score of 1 is defined as borderline, and a score of 2 or more defines abnormal/caseness (Goodman 2011). These values were for logistic regression further dichotomised into normal versus borderline/abnormal. We decided to restrict the definition of mental health problems to those exhibiting both symptoms of problems as measured by the SDQ symptom score, and additionally showing indications of overall distress and social impairment. Thus a new variable was made that summed the dichotomous symptom score and the dichotomous impact score (Goodman 2011). The resulting variable was further dichotomised. To qualify as having a mental health problem, the participants thus had to be borderline or abnormal for both the total symptom score and the impact score. The Cronbach’s alpha for the 19 questions of SDQ total symptoms excluding the headache question was found to be 0.78.

*Coping strategies* were assessed by the scenario: “What do you do/what happens when you are burdened by painful thoughts and feelings?” We used seven items (Table 1), each with three categorical answers (0 = not true, 1 = somewhat true and 2 = certainly true). We divided coping into four internal (ICS1, ICS2, ICS3 and ICS4) and three external (ECS1, ECS2 and ECS3) coping strategies (see Table 1). These were treated as independent variables. The correlation between the coping variables was tested by Pearson’s r and found not to be substantial, ranging from 0 to 0.28. Confounders adjusted for in the analyses were gender, grade, socioeconomic status, living with both parents or not, subjective school-related stress and nation of origin (separated as western or non-western). For the variable coping strategies, missing data ranged between 937 and 1459 of the total number (19,985).

**Table 1:** List of coping strategies [internal (ICS) and external (ECS) coping strategies]

		N (%)
ICS 1—Keep painful thoughts and feelings inside	No	15,875 (84.5)
	Yes	2919 (15.5)
ICS 2—Work more with other things to avoid thinking bad thoughts	No	14,106 (75.3)
	Yes	4615 (24.7)
ICS 3—Using abusive substances when having bad thoughts or feelings	No	17,809 (95.8)
	Yes	772 (4.2)
ICS 4—Try to talk oneself out of problems	No	16,574 (89.0)
	Yes	2044 (11.0)
ECS 1—Visit health care service when having bad thoughts or feelings	No	18,225 (98.4)
	Yes	301 (1.6)
ECS 2—Speak with family when having bad thoughts or feelings	No	14,256 (75.6)
	Yes	4600 (24.4)
ECS 3—Speak with friends when having bad thoughts or feelings	No	9355 (49.1)
	Yes	9693 (50.9)

No is defined as either not true or somewhat true, whereas yes is equivalent to certainly true

### Ethics

Participation was voluntary, all questionnaires were anonymous, and based on individual informed consent. Pupils in secondary schools had parental consent. The health survey was conducted after approval from the Regional Ethics Committee.

### Statistical analyses

All preliminary analyses were performed by the Statistical Package for Social Sciences (SPSS) version 22.0. Confirmatory factor analysis (CFA) operations were then conducted using maximum likelihood (ML) estimation by means of Analysis of Moment Structures (AMOS version 22) (Arbuckle 2013).

Multinomial logistic regression analyses were used to estimate the associations between chronic headache and mental health groups (CH, CHMH, MH) and the independent variables as compared to the control group. The analyses were stratified by health group. All analyses were controlled for age, gender, socioeconomic status, living with both parents or not, subjective school-related stress and nation of origin. Due to a complex sampling design with county and class as unit, logistic regression analyses were performed using the SPSS module, complex samples, which corrects estimates of standard errors accounting for sampling design (Osborne 2011). Odds ratio [with 95 % confidence interval (CI)] was used to estimate outcome, for multiple comparisons Bonferroni corrected p values (p < 0.0017) were used to avoid the risk of mass significance. Cases were removed if at least one variable was missing (listwise deletion). The significance level was set to p < 0.05 and effect estimates reported Beta with SE. All variables were checked for multicollinearity. Tolerance should not be above 0.10 and VIF should be below 10 (Pallant 2010). These assumptions were not violated for any variables in the analyses.

To evaluate the coping strategy profiles, we performed CFAs (Bollen 2014) using data from each of the different subgroups. The analyses were run by means of ML estimation.

As the χ^2^ has been shown to be problematic for assessing model fit in large samples (Byrne 2013; Cheung and Rensvold 2002; Hirschfeld and von Brachel 2014; Hooper et al. 2008), model fit was primarily assessed using the root mean square error of approximation (RMSEA) with values of 0.08, 0.05 and 0, and the comparative fit index (CFI), with values 0.90, 0.95 and 1.0 demonstrating reasonable, close and exact fit, respectively.

Invariance testing was conducted by multi-group CFAs using ML estimation in AMOS 22. This method employs successive analyses where constraints to the models are added consecutively. We used the baseline, unconstrained model, with one factor loading constrained to unity. Good model fit at this stage and significant factor loadings is indicative of configural invariance (Hirschfeld and von Brachel 2014). In addition, the weak (metric) model was used. Invariance at this level implies that the regression slopes are invariant across groups and implies that the same latent variables are being measured across groups. ΔCFI was used with a cut-off ≤0.01 indicating invariance between subgroups (Byrne 2013; Hirschfeld and von Brachel 2014; Hooper et al. 2008; Meade et al. 2008).

## Results

### Prevalence

To test for representativity of the sample, the prevalence of some of the control variables were compared to national averages for 2002 (Statistics Norway 2015). The results indicated that there were identical prevalence of males and females (M = 51 %; F = 49 %) in both sample and national values; nearly identical prevalence of those with Norwegian national identity (84.7 % nationwide versus 84.6 in this sample); while prevalence of those living with both parents was slightly higher in the sample (67.5 %) than the nationwide values of 62.1 %.

3.7 % of participants (n = 717) met the criteria for CH, 1.1 % (n = 212) for CHMH and 5.5 % (n = 1049) for MH, whereas 89.7 % are in the control group (N = 17,143) (thus a total N = 19,121 after missing values for disease criteria were removed). Of the 929 adolescents with chronic headaches, 23 % thus had comorbid mental health problems compared with a 5.8 % prevalence of mental health problems among those without chronic headache. The relative risk (RR) of having chronic headaches when also having mental health problems was 4.2 (95 % CI: 3.6–4.8) while the RR of having mental health problems when also having chronic headaches was 4.0 (95 % CI: 3.5–4.5). Tables 2 and 3 show the prevalence of the demographic variables and the coping strategies in the three groups: CH, CHMH and MH.

**Table 2:** Comparison of coping strategies [internal (ICS) and external (ECS) coping strategies] between headache and mental health groups

		Control N (%)	CH N (%)	CHMH N (%)	MH N (%)
ICS 1—Keep painful thoughts and feelings inside	No	14,202 (86.8)^a^	510 (75.8)^c^	94 (46.5)^d^	643 (63.9)^b^
	Yes	2155 (13.2)^a^	163 (24.2)^c^	108 (53.5)^d^	363 (36.1)^b^
ICS 2—Work more with other things to avoid thinking bad thoughts	No	12,475 (76.3)^a^	464 (69.8)^b^	131 (65.8)^b^	668 (68.0)^b^
	Yes	3868 (23.7)^a^	201 (30.2)^b^	68 (34.2)^b^	314 (32.0)^b^
ICS 3—Using abusive substances when having bad thoughts or feelings	No	15,727 (97.0)^a^	598 (92.0)^c^	160 (79.2)^d^	862 (86.5)^b^
	Yes	489 (3.0)^a^	52 (8.0)^c^	42 (20.8)^d^	134 (13.5)^b^
ICS 4—Try to talk oneself out of problems	No	14,756 (90.8)^a^	520 (78.8)^b^	124 (61.4)^c^	745 (74.9)^b^
	Yes	1502 (9.2)^a^	140 (21.2)^b^	78 (38.6)^c^	249 (25.1)^b^
ECS 1—Visit health care service when having bad thoughts or feelings	No	15,999 (98.8)^a^	629 (96.3)^b^	183 (91.5)^c^	949 (95.6)^b,c^
	Yes	199 (1.2)^a^	24 (3.7)^b^	17 (8.5)^c^	44 (4.4)^b,c^
ECS 2—Speak with family when having bad thoughts or feelings	No	12,308 (74.9)^a^	519 (77.8)^a,c^	171 (85.5)^b,c^	873 (87.2)^b^
	Yes	4123 (25.1)^a^	148 (22.2)^a,c^	29 (14.5)^b,c^	128 (12.8)^b^
ECS 3—Speak with friends when having bad thoughts or feelings	No	8004 (48.2)^a^	320 (47.6)^a^	128 (64.0)^b^	627 (62.2)^b^
	Yes	8592 (51.8)^a^	352 (52.4)^a^	72 (36.0)^b^	381 (37.8)^b^

No is defined as either not true or somewhat true, whereas yes is equivalent to certainly true

Values in the same row and subtable not sharing the same superscript are significantly different at p < 0.05 in the two-sided test of equality for column proportions. Tests assume equal variances (tests are adjusted for all pairwise comparisons within a row of each innermost subtable using the Bonferroni correction)

**Table 3:** Prevalence (%) of background variables and independent variables in the groups: control group CH, MH and CHMH

	Control group	CH	CHMH	MH
Sex				
Boy	52.0^a^	30.4^c^	31.9^c^	41.8^b^
Girl	48.0^a^	69.6^c^	68.1^c^	58.2^b^
Grade				
8th grade LSS	17.3^a^	16.8^a,c^	10.0^b,c^	11.9^b^
9th grade LSS	15.7^a^	16.2^a^	19.0^a^	15.1^a^
10th grade LSS	14.5^a^	14.1^a^	13.3^a^	12.8^a^
1st grade USS	24.2^a^	27.1^a,b^	29.9^a,b^	27.8^b^
2nd grade USS	16.6^a^	14.7^a^	18.0^a^	18.2^a^
3rd grade USS	11.7^a^	11.0^a^	10.0^a^	14.2^a^
How well off is your family?				
Very good	13.3^a^	14.5^a^	11.8^a^	11.1^a^
Good	48.9^a^	41.7^c^	27.0^b^	32.0^b^
Medium	31.2^a^	30.6^a^	37.9^a,b^	37.0^b^
Not very good	5.4^a^	9.7^b^	12.3^b^	13.6^b^
Poorly	1.1^a^	3.4^c^	10.9^b^	6.3^b^
Lives with both parents				
Yes	69.1^a^	60.4^c^	51.2^b,c^	53.3^b^
No	30.9^a^	39.6^c^	48.8^b,c^	46.7^b^
How stressed are you of school work?				
Not at all	11.5^a^	7.5^b^	8.6^a,b^	7.2^b^
A little	47.1^a^	28.5^b^	18.6^c^	23.1^b,c^
Pretty much	28.4^a^	33.9^b^	22.9^a^	33.6^b^
Very much	13.0^a^	30.1^b^	50.0^c^	36.1^b^
Nation of origin				
Western country (incl. Norway)	94.9^a^	93.6^a,b^	93.9^a,b^	91.3^b^
Asia/Afrika/Latin America	5.1^a^	6.4^a,b^	6.1^a,b^	8.7^b^

Values in the same row and subtable not sharing the same superscript are significantly different at p < 0.05 in the two-sided test of equality for column proportions. Tests assume equal variances (tests are adjusted for all pairwise comparisons within a row of each innermost subtable using the Bonferroni correction)

*LSS* lower secondary school, *USS* upper secondary school

### Chronic headaches without mental health problems

There was a relationship between having chronic headaches, and using the internal coping strategies of keeping troubles inside, and using abusive substances when bad thoughts and feelings create pressure (Table 4).

**Table 4:** Multivariate logistic regression analysis examining the association between the three groups: CH, CHMH, MH, and internal (ICS) and external (ECS) coping strategies

Coping strategy (reference “not true”)	CH vs control OR (95 % CI)	CHMH vs control OR (95 % CI)	MH vs control OR (95 % CI)
N	15,828	15,463	16,487
ICS 1—Keep painful thoughts and feelings inside (reference “not true”)	1.22 (1.15–1.29)*	2.96 (2.81–3.12)*	1.65 (1.58–1.73)*
ICS 2—Work more with other things to avoid thinking bad thoughts	1.07 (1.05–1.08)*	0.80 (0.76–0.84)*	0.93 (0.89–0.98)
ICS 3—Using abusive substances when having bad thoughts or feelings	1.46 (1.42–1.49)*	2.39 (2.25–2.53)*	1.90 (1.86–1.95)*
ICS 4—Try to talk oneself out of problems	1.32 (1.29–1.37)*	2.00 (2.19–2.61)*	1.77 (1.73–1.80)*
ECS 1—Visit health care service when having bad thoughts or feelings	1.47 (1.34–1.61)*	2.39 (2.19–2.61)*	1.72 (1.65–1.79)*
ECS 2—Speak with family when having bad thoughts or feelings	0.83 (0.80–0.86)*	0.68 (0.64–0.73)*	0.70 (0.66–0.74)*
ECS 3—Speak with friends when having bad thoughts or feelings	0.82 (0.78–0.85)*	0.69 (0.67–0.71)*	0.66 (0.64–0.68)*

The three groups are all compared to the control group having neither CH or MH. Analyses done with complex samples

Cell values are odds ratios with 95 % confidence intervals

Controlled for sex, grade, socio-economic status, lives with both parents, school-related stress and nation of origin

*ICS* internal coping strategy, *ECS* external coping strategy

* p ≤ 0.0017 significance limits based on pre-decided limits which are corrected for 28 multiple comparisons by dividing p = 0.05 by 30

The CH group also tended to use the internal coping strategy of talking themselves out of their problems, and the external coping strategy of visiting health care services, more so than the control group. The CH subjects were less likely to use the external coping strategies of speaking with friends or family compared with the control group. The rank order of odds ratios for coping strategies used more by the CH group was: visit health care services > using abusive substances > talk oneself out of problems > keeping painful thoughts or feelings inside.

### Chronic headaches with simultaneous mental health problems

The CHMH subjects were more than two times more likely to use the internal coping strategies of keeping painful thoughts or feelings inside, using abusive substances and talking themselves out of their problems than the control group (Table 4). The probability was two times higher for using the external coping strategy of visiting health care services when bad thoughts and feelings were present among the CHMH group, compared with the control group. In comparison with the CH group, the CHMH group was significantly more likely to use the internal coping strategies of keeping painful thoughts or feelings inside, using abusive substances and talking oneself out of problems. The CHMH group was also less likely to use the internal coping strategy of working more with other things. The CHMH group tended to use the external coping strategy of speaking with family less compared with the CH group (not significant) (Table 4). The rank order of odds ratios for coping strategies used more by this group was: keep painful thoughts or feelings inside > using abusive substances > visit health care service > talk oneself out of problems. Speaking with others (both friends and family) and doing other things were little used strategies in this group.

### Mental health problems without chronic headaches

The MH group used the internal coping strategy of keeping painful thoughts or feelings inside to a greater extent, compared with the control group (Table 4). The odds of using abusive substances as a coping strategy increased by a factor of 1.9 in the MH group, compared with the control group. There was a tendency among the MH group to use the external coping strategies of talking themselves out of problems, and seeking help from health care services when bad thoughts and feelings were present, compared to the control group. The rank order of odds ratios used more by this group was: using abusive substances > talk oneself out of problems > visit health care service > keep painful thoughts or feelings inside. Speaking with others (friends and family) was also here a less used strategy than in the control population.

### Effects of demographic variables on main outcomes (Table 3)

Proportion of adolescents classified as CH or CHMH significantly decreased with increasing age, whereas proportions with MH alone increased with age. Female gender was a significant factor associated with more frequent caseness in all three outcome groups. Living situation (i.e. living with both parents as opposed to not) was a significant factor associated with less frequent caseness in all three outcome groups. Socioeconomic status was a significant factor associated with more frequent MH, CH and CHMH with decreasing family income. Experienced school stress was a significant factor associated with more frequent MH, CH and CHMH. Having a non-Western nation of origin was only significantly associated with higher MH. These demographic parameters were used as possible confounders and were adjusted for in main outcome analyses.

### Coping strategy profiles in the different health groups

**Fig. 1:**
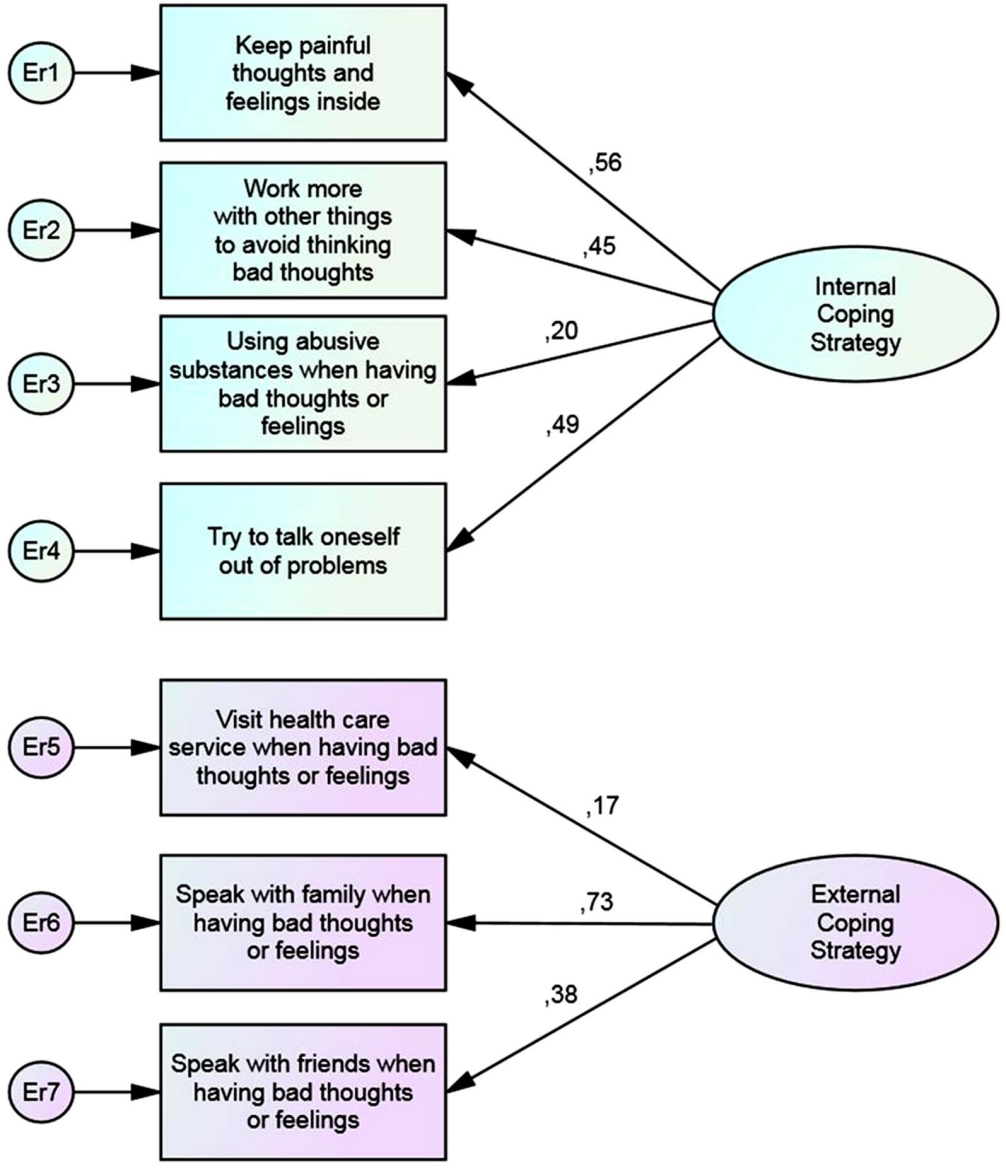
Comparison of standardized factor loadings of individual coping strategies on latent internal (ICS) and external (ECS) coping strategies in the unconstrained model

The results of the Confirmatory Factor Analysis (CFA) for each group is shown in Fig. 1, which presents the standardized coefficients of the unconstrained model for the entire population. Table 5 presents the values for each of the subgroups,MH, CH and CHMH. Using abusive substances consistently loaded less on the latent variable “internal coping strategies” than the other three related variables. The relative weight of these remaining three internal strategies were much closer to each other for all groups. Keeping painful thoughts and feelings inside was most strongly related to being in the CH group. Among the factor loadings related to the latent variable, external coping strategy, the strongest loading for all groups was towards speaking with family. The groups differed substantially in loadings of the individual strategies. The model showed moderate fit. The differences in the pattern of internal and external strategies used by the four groups were significant as tested by invariance testing (data not shown) and using the value of <0.01 as cut-off for ∆CFI.

**Table 5:** Standardized factor loadings for all seven items of coping strategies in a two factor model for each population subgroup (N = 19,121)

	Control	CH	CHMH	MH
N	17,143	717	212	1049
ICS 1—Keep painful thoughts and feelings inside	0.53	0.60	0.48	0.44
ICS 2—Work more with other things to avoid thinking bad thoughts	0.47	0.52	0.50	0.43
ICS 3—Using abusive substances when having bad thoughts or feelings	0.14	0.15	0.12	0.13
ICS 4—Try to talk oneself out of problems	0.45	0.48	0.42	0.50
ECS 1—Visit health care service when having bad thoughts or feelings	0.18	0.30	0.23	0.28
ECS 2—Speak with family when having bad thoughts or feelings	0.66	0.72	1.40	0.82
ECS 3—Speak with friends when having bad thoughts or feelings	0.41	0.40	0.13	0.29
χ^2^	2870			
CFI	0.615			
RMSEA	0.051			

*ICS* internal coping strategy, *ECS* external coping strategy, *CFI* comparative fit index, *RMSEA* root mean square error of approximation

## Discussion

The current study examined the relationship between chronic headaches and coping strategies in adolescents. Chronic headaches among youth were associated with a higher risk of having mental health problems and vice versa. Our results suggest that different coping strategies were used among the four groups. Mental health impact is similar in groups with mental health problems whether or not they have chronic headaches, while youth without mental health problems reported lower impact, closer to that of the control group.

The present study is based on a large sample size and the response rate was high (82 %). Akershus county in the south east part of Norway, where the study took place is representative for the nation as a whole compared to national data (Statistics Norway 2015). The questionnaire was aimed at a broad description of health in youth and was not specific to the disease category of chronic headaches. Therefore, respondents could not know the purpose of the present study, namely mapping chronic headache disorders in adolescents. Chronic headache was measured with one simple question about several complaints during the past 6 months where headache was one of the complaints. Based on this question, we do not get information about important aspects of the headache: duration of the headache and how strong the headache is. Furthermore, the quantification (number of days of headache per month) which is an important criterion in the classification of headache, is imprecise here. Thus we cannot claim to have precise clinical headache diagnoses as basis for our study. On the other hand, as stated above, few if any headache studies have focused on coping strategies in the detail that we have here. The criteria used to define the presence of mental health problems were strengthened to require the simultaneous presence of both abnormal or borderline symptom score and impact in SDQ (Goodman 2001). Several studies show that SDQ is a useful and valid tool for identifying mental health complications among children and adolescents (Goodman 2011; Mathai et al. 2004). We have therefore chosen to use this tool though there is so far no consensus on the optimal instrument. Studies are ongoing which search for improved instruments for assessing relevant psychological factors in chronic headache such as the “stagnation scale” (Innamorati et al. 2015). As described in the introduction, the various axes for measuring coping strategies are thus under debate. Though our coping strategy questions are not validated against other measures, we suggest that they may nevertheless be useful as defined. This study is based on self-report, and there is no clinical validation of the answers. We have no information concerning use of medication in connection with headaches, which may be of importance in relation both to contact with health services and other internal versus external coping strategies. On the other hand, it is more difficult to assign a headache diagnosis in children, partly because of the paediatric age (Seshia et al. 2010) and different diagnostic methods that have been used, making comparison difficult (Lipton et al. 2011).

The 6-month prevalence of chronic headaches (3.7 %) was considerably higher than that found in other studies among young people (Seshia et al. 2010). Possible explanations for the discrepancy, as compared with our study, may be: (1) different definitions of chronic headache, (2) variations in measuring instruments, (3) variations in the specified time frame for the headache, and (4) older age group in our study (13–19 years). Our data are based on self-evaluation which may also contribute to this difference. In addition, smaller sample sizes may give more uncertain estimates. We found that the relative risk (RR) of having chronic headaches when having mental health problems and vice versa was about 4. However, a cross-sectional study such as ours cannot answer the “chicken and egg” issue, thus our results underline the need for prospectively designed studies with emphasis on prognosis and etiological factors.

The prevalence of mental health problems among those with chronic headaches was found to be 23 %. A study by Wang and colleagues (Wang et al. 2007) reported psychiatric disorders in almost half of the 121 Taiwanese school children aged 12–14 years with chronic headaches. Other studies have found psychiatric disorders in 64–90 % of patients with chronic headaches (Puca 2000; Verri et al. 1998). Differences across studies in the prevalence of psychiatric comorbidity in patients with chronic headaches may be due to the measuring instrument used to define psychological functioning. However, compared with controls without chronic headache, our data show that the prevalence of mental health problems in youth with chronic headache is high. We have focused on a group of adolescents, who, in addition to having chronic headache complaints, also have psychological problems. Few studies have examined the differences in coping strategies in adolescents with chronic headaches with or without comorbid psychiatric disorders. Previous studies have found that patients with chronic headache show an overall avoidance coping pattern (Rollnik et al. 2001), associated with increased psychological problems (Seiffge-Krenke 2000). It is likely that adolescents are even more inclined to use less mature coping strategies when having chronic headaches with comorbid mental health problems. This is in accordance with our study.

The CHMH group in the present study used internal coping strategies, to a larger degree than the two other groups. These findings are similar to Jorgensen and Dusek (Jorgensen and Dusek 1990), where less psychologically adjusted adolescents used less mature coping strategies like alcohol use and minimizing the problem to a greater degree. Ebata and Moos (Ebata and Moos 1991) had a similar finding in a longitudinal study of life stressors, social resources and coping among adolescents aged 12–18, where depressed adolescents and adolescents with conduct disorder used more avoidance coping mechanisms than healthy adolescents. In contrast, Murberg and Bru (Murberg and Bru 2005) did not find an effect of problem-focused coping strategies on symptoms of depression among Norwegian adolescents. Lanzi and colleagues (Lanzi et al. 2001) found that headache sufferers internalized their feelings, which to some extent may seem to support our findings. The CH group was more likely to use other strategies, compared to the CHMH group. According to Compas and colleagues (Compas et al. 2001), distraction decreased the levels of distress and intrusive thoughts. Thus our data suggests that the presence of mental health problems in adolescents with chronic headaches make the youth less able to distract themselves from troubled thoughts.

External coping strategies were used less commonly in the CHMH group compared to the control group with the exception of visiting health care services. Visiting health care services can be considered an external coping strategy, and contradicts studies saying that adolescents struggling with mental health problems show an overall coping strategy of avoidance (Chan 1995; Seiffge-Krenke 2000; Ebata and Moos 1991). This may, however, reflect the low threshold in Norway for visiting health units, since they are placed in or near schools. Speaking with family and friends were little used coping strategies in all groups, especially in the groups with mental health problems, and may reflect social isolation in adolescents struggling with mental health problems with or without chronic headaches. According to Martin and Theunissen (Martin and Theunissen 1993), adults with chronic headaches score significantly lower on social support, compared to non-headache subjects, which can be an indicator that chronic headache sufferers are less able to seek support from family or peers. The study by Murberg and Bru (Murberg and Bru 2005) found decreased levels of symptoms of depression in Norwegian adolescents that seek parental support in stressful situations. According to the latter study, the importance of the external coping strategies seeking parental or friend support are essential for mental health among adolescents. Our data suggests it may be even more important when having the additional burden of a chronic headache.

Categorising coping strategies into two dimensions, e.g. internal versus external coping, has been criticized (Holen et al. 2012). It has been suggested that the studies do not adequately distinguish between the types of emotional coping strategies. Some found that a strategy based on emotion-focused coping is only related to risk if the use of other coping strategies is limited, others suggest that children who are flexible in their use of coping strategies have better mental health outcomes. The most recent studies on coping in children suggest that flexible use of three or more coping strategies may be advantageous (Holen et al. 2012). This study did indicate however, that coping strategy profiles used were significantly different between all groups.

## Conclusion

In this study we have found that adolescents with chronic headaches show more frequent mental health problems than those without chronic headaches. The group of adolescents having both chronic headaches and mental health problems appear to be the most vulnerable population. Compared to adolescents without mental health problems, adolescents with chronic headaches that have simultaneous mental health problems, to a greater extent use internal coping strategies and to a lesser degree seek support in their social networks. Efforts should be made by school and health services, and in local communities to promote the use of external coping strategies in high-risk groups having both chronic headache and mental health problems. Exactly how this could be done requires further prospective, longitudinal follow-up studies of such issue-adapted treatment including a focus both on headache load, psychopathology and coping.
